# Product of the Hen

**Published:** 1883-09

**Authors:** 


### PRODUCT OF THE HEN.

The hen has in her ovaries in round numbers, more than 600 egg germs, which develope gradually and are successively laid. Of these 600 the hen will lay twenty in her first year, 135 in her second, and 114 in the third. In each one of the following four years the number of eggs laid will be diminished by twenty, and in her ninth year she will lay at most, ten eggs. In order to obtain from them sufficient product to cover the expense of alimentation, they should not be allowed to live over four years.—*Annales de la Sociedad Odontologica*, Havana.

